# Publisher Correction: TNF and type I interferon crosstalk controls the fate and function of plasmacytoid dendritic cells

**DOI:** 10.1038/s41590-025-02293-6

**Published:** 2025-09-05

**Authors:** Rebeca Arroyo Hornero, Raul A. Maqueda-Alfaro, Miguel A. Solís-Barbosa, Rebecca A. Leylek, Olin Medina Chavez, Olivia M. Martinez, Andres Gottfried-Blackmore, Juliana Idoyaga

**Affiliations:** 1 Department of Microbiology and Immunology, Stanford University School of Medicine, Stanford, CA USA; 2 Immunology Program, Stanford University School of Medicine, Stanford, CA USA; 3 Department of Pharmacology, University of California San Diego School of Medicine, La Jolla, CA USA; 4 Department of Surgery, Division of Abdominal Transplantation, Stanford University School of Medicine, Stanford, CA USA; 5 Department of Medicine, Division of Gastroenterology, University of California San Diego School of Medicine, La Jolla, CA USA; 6 Gastroenterology Section, Veterans Affairs San Diego Healthcare System, La Jolla, CA USA; 7 Department of Molecular Biology, University of California San Diego School of Biological Sciences, La Jolla, CA USA

**Keywords:** Dendritic cells, Antigen-presenting cells, Interferons, Chromatin remodelling

Correction to: *Nature Immunology* 10.1038/s41590-025-02234-3, published online 12 August 2025.

In the version of this article initially published, due to processing errors, in Fig. 4p, the TCF4 graph was a duplicate of the adjacent IRF8 graph and has now been replaced, while in Fig. 2f, h, i, labels reading “C1, C2, C3” appeared originally as “#1, #2, #3.” The figures have been updated in the HTML and PDF versions of the article.

