# Supplementary material for: TNF and type I interferon crosstalk controls the fate and function of plasmacytoid dendritic cells
Source: Nat Immunol. 2025 Aug 12;26(9):1540–52. doi: 10.1038/s41590-025-02234-3 (PMC12396960; doi:10.1038/s41590-025-02234-3)
Supplement: Supplementary file 1 — Reporting Summary [file 41590_2025_2234_MOESM1_ESM.pdf]

nature portfolio

Corresponding author(s): Idoyaga, Juliana

Last updated by author(s): 7/23/2025

Reporting Summary

Nature Portfolio wishes to improve the reproducibility of the work that we publish. This form provides structure for consistency and transparency in reporting. For further information on Nature Portfolio policies, see our Editorial Policies and the Editorial Policy Checklist.

Statistics

For all statistical analyses, confirm that the following items are present in the figure legend, table legend, main text, or Methods section.

n/a	Confirmed	
☐	☒	The exact sample size (*n*) for each experimental group/condition, given as a discrete number and unit of measurement
☐	☒	A statement on whether measurements were taken from distinct samples or whether the same sample was measured repeatedly
☐	☒	The statistical test(s) used AND whether they are one- or two-sided<br>*Only common tests should be described solely by name; describe more complex techniques in the Methods section.*
☒	☐	A description of all covariates tested
☐	☒	A description of any assumptions or corrections, such as tests of normality and adjustment for multiple comparisons
☐	☒	A full description of the statistical parameters including central tendency (e.g. means) or other basic estimates (e.g. regression coefficient) AND variation (e.g. standard deviation) or associated estimates of uncertainty (e.g. confidence intervals)
☐	☒	For null hypothesis testing, the test statistic (e.g. *F*, *t*, *r*) with confidence intervals, effect sizes, degrees of freedom and *P* value noted<br>*Give P values as exact values whenever suitable.*
☒	☐	For Bayesian analysis, information on the choice of priors and Markov chain Monte Carlo settings
☒	☐	For hierarchical and complex designs, identification of the appropriate level for tests and full reporting of outcomes
☐	☒	Estimates of effect sizes (e.g. Cohen's *d*, Pearson's *r*), indicating how they were calculated

Our web collection on statistics for biologists contains articles on many of the points above.

Software and code

Policy information about availability of computer code

Data collection	Flow cytometry data was acquired using BD FACS Diva software (v8.01) or CYTEK SpectroFlo (v3.3.0). RNA Sequencing data were obtained using Illumina novaseq6000 platform.
Data analysis	Flow cytometry data was analyzed using FlowJo software (v.10.10.0). Sequencing data was analyzed using the following programs: Seurat (v.5.0.2), Signac (v.1.12.9004), R (v.4.2.3), GSEA (v.4.2.3), chromVAR (v.1.22.1), Monocle3 (v.2.28.0), Slingshot (v.2.8.0), Harmony (v.1.2.0), viridis (v.0.6.5), ggplot2 (v.3.5.0), scCustomize (v.2.1.2), Triwise (v.0.99.5), MACS2 (v.2.2.9.1), DESEq2 (v.3.14), Pheatmap (v. 1. 0.12). Bulk RNAseq data was aligned using STAR (2.7.3a), and bulk ATACseq data was processed using (nfatachttps://nf-co.re/atacseq/2.1.2). CyTOF data was analyzed using FlowJo (v10.10.1), R (v.4.3.1), Scaffold (v0.1) (https://github.com/nolanlab/scaffold.git), Premessa (v.0.3.4), Marker Enrichment Modeling (v.0.1.1) and MATLAB (v2023b). Graphing and Statistical analysis were performed on GraphPad Prism 10 (GraphPad Software, Inc.).

For manuscripts utilizing custom algorithms or software that are central to the research but not yet described in published literature, software must be made available to editors and reviewers. We strongly encourage code deposition in a community repository (e.g. GitHub). See the Nature Portfolio guidelines for submitting code & software for further information.

Data

Policy information about availability of data

All manuscripts must include a data availability statement. This statement should provide the following information, where applicable:

- Accession codes, unique identifiers, or web links for publicly available datasets
- A description of any restrictions on data availability
- For clinical datasets or third party data, please ensure that the statement adheres to our policy

Data generated in this study have been deposited in NCBI GEO under accession numbers: GSE267100 (GRCh38 reference genome); GSE267099 (GRCh38 reference genome); GSE267174 (GRCh38 reference genome); GSE266889 (hg19 reference genome), GSE269411 (hg19 reference genome); and GSE279911 (hg19 reference genome). Publicly available CITE-seq (EGAS00001005271) was download from (https://explore.data.humancellatlas.org/projects/5bd01deb-01ee-4611-8efd-cf0ec5f56ac4/project-matrices). Publicly available SMART-seq2 (E-MTAB-8498) dataset was downloaded from (https://explore.data.humancellatlas.org/projects/67a3de09-45b9-49c3-a068-ff4665daa50e/project-metadata). Source data are provided with this paper.

Research involving human participants, their data, or biological material

Policy information about studies with human participants or human data. See also policy information about sex, gender (identity/presentation), and sexual orientation and race, ethnicity and racism.

Reporting on sex and gender	When provided, information on donor age and sex is reported in the Methods (see below) or in the supporting data (Supplementary Table 6). Samples were not selected based on sex, age (beyond the range described above), race, or other individual characteristics.
Reporting on race, ethnicity, or other socially relevant groupings	This manuscript did not consider race and ethnicity during data analyses.
Population characteristics	Healthy donors (buffy coats and blood drawn) between 20-40 and over 65 years old, without signs of infection for at least 2 weeks.
Recruitment	De-identified blood (collected using EDTA-coated tubes; BD Biosciences) and buffy coats from healthy adults (20–40 years) and elderly donors (>65 years; Supplementary Table 6) were obtained through local lab-led blood donation efforts following Stanford University and UCSD guidelines, or from the Stanford Blood Center. Donors provided informed consent under protocols approved by the Institutional Review Boards (IRBs) of Stanford University and UC San Diego, and did not receive compensation.
Ethics oversight	De-identified blood and buffy coats were obtained following Stanford University and UCSD guidelines. Donors provided informed consent under protocols approved by the Institutional Review Boards (IRBs) of Stanford University and UC San Diego.

Note that full information on the approval of the study protocol must also be provided in the manuscript.

Field-specific reporting

Please select the one below that is the best fit for your research. If you are not sure, read the appropriate sections before making your selection.

☒ Life sciences ☐ Behavioural & social sciences ☐ Ecological, evolutionary & environmental sciences

For a reference copy of the document with all sections, see nature.com/documents/nr-reporting-summary-flat.pdf

Life sciences study design

All studies must disclose on these points even when the disclosure is negative.

Sample size	No sample-size calculations were performed, but sample sizes are similar to those reported in previous calculations (Leylek, et al. Cell Reports 2019; 2020; Alculumbre et al., Nat. Immunol 2018; Palucka et al., PNAS, 2005; Alcantara-Hernandez et al., Immunity 2017).
Data exclusions	No data were excluded. For sequencing data, pre-established criteria for single-cell exclusion, i.e., low number of unique genes, abnormally high read count, and high mitochondrial gene content, was used.
Replication	All assays were repeated at least twice with multiple blood donors. All attempts of replication were successful.
Randomization	Blood samples were randomly allocated across experiments.
Blinding	Investigators were not blinded because no assay where it would be necessary was performed. We performed quantitative measurements and unbiased computational analyzes.

Reporting for specific materials, systems and methods

We require information from authors about some types of materials, experimental systems and methods used in many studies. Here, indicate whether each material, system or method listed is relevant to your study. If you are not sure if a list item applies to your research, read the appropriate section before selecting a response.

Materials & experimental systems

n/a	Involved in the study	
☐	☒	Antibodies
☒	☐	Eukaryotic cell lines
☒	☐	Palaeontology and archaeology
☒	☐	Animals and other organisms
☒	☐	Clinical data
☒	☐	Dual use research of concern
☒	☐	Plants

Methods

n/a	Involved in the study	
☒	☐	ChIP-seq
☐	☒	Flow cytometry
☒	☐	MRI-based neuroimaging

Antibodies

Antibodies used

Species reactivity Target Color Clone Catolog number Company Identifier Dilution
Anti-human AXL (clone 108724) AF488 R&D Systems Cat# FAB154G; RRID: AB_2714170; 1:50
Anti-human AXL (clone DS7HAXL) PE-Cy7 Thermo Fisher Cat# 25-1087-42; RRID:AB_2723959; 1:100
Anti-human BDCA1 (clone L161) APC-Cy7 Biolegend Cat# 331520; RRID:AB_10644008; 1:100
Anti-human BDCA2 (clone 201A) FITC Biolegend Cat# 354208; RRID:AB_2561364; 1:50
Anti-human BDCA3 (clone M80) BV785 Biolegend Cat# 344116; RRID:AB_2572194; 1:100
Anti-human BDCA4 (clone 12C2) PerCPCy5.5 Biolegend Cat# 354510; RRID:AB_2561558; 1:100
Anti-human BDCA4 (clone 12C2) APC Biolegend Cat# 354506; RRID:AB_11219600; 1:400
Anti-human BDCA4 (clone 12C2) BV510 Biolegend Cat# 354515; RRID:AB_25630741:200
Anti-human BDCA4 (clone M80) BV785 Biolegend Cat# 344116; RRID:AB_2572194; 1:100
Anti-human CCR2 (clone K036C2) APC Biolegend Cat# 357207; RRID:AB_2562239; 1:50
Anti-human CCR7 (clone G043H7) PE-Cy7 Biolegend Cat# 353226; RRID:AB_11125576; 1:50
Anti-human CD11c (clone Bu15) Alexa700 Biolegend Cat# 337220; RRID:AB_2561502; 1:200
Anti-human CD123 (clone 7G3) BUV395 BD Biosciences Cat# 564195; RRID:AB_2714171; 1:100
Anti-human CD123 (clone 6H6) FITC Biolegend Cat# 306014; RRID:AB_2124259; 1:200
Anti-human CD123 (clone 6H6) PE Biolegend Cat# 306006; RRID:AB_314580; 1:400
Anti-human CD123 (clone 6H6) PE-Cy7 Biolegend Cat# 306009; RRID:AB_493576; 1:200
Anti-human CD123 (clone 6H6) PE/Dazzle 594 Biolegend Cat# 306034; RRID:AB_2566450; 1:200
Anti-human CD14 (clone M5E2) BV650 Biolegend Cat# 301836; RRID:AB_11204241; 1:100
Anti-human CD14 (clone M5E2) BV785 Biolegend Cat# 301840; RRID:AB_2563425; 1:200
Anti-human CD16 (clone 3G8) BV650 Biolegend Cat# 302042; RRID:AB_11125578; 1:200
Anti-human CD19 (clone HIB19) PerCP Cy5.5 Biolegend Cat# 302230; RRID:AB_2275547; 1:200
Anti-human CD19 (clone HIB19) PB Biolegend Cat# 302232; RRID: AB_2073118; 1:50
Anti-human CD20 (clone 2H7) PerCP Cy5.5 Biolegend Cat# 302326; RRID:AB_893285; 1:200
Anti-human CD20 (clone 2H7) PB Biolegend Cat# 302328; RRID AB_1595435; 1:200
Anti-human CD25 (clone BC96) PE Biolgend Cat# 302606; RRID:AB_314275; 1:200
Anti-human CD3 (clone UCTH1) PECy7 Biolegend Cat# 300419; RRID:AB_439781; 1:200
Anti-human CD3 (clone UCTH1) PerCP Cy5.5 Biolegend Cat# 300430; RRID:AB_893299; 1:200
Anti-human CD3 (clone UCTH1) PB Biolegend Cat # 300431; RRID: AB_1595437; 1:100
Anti-human CD33 (clone WM53) BV650 Biolegend Cat# 303430; RRID:AB_2650933; 1:25
Anti-human CD33 (clone WM53) PE Biolegend Cat# 303403; RRID:AB_314348; 1:100
Anti-human CD33 (clone WM53) PE-Cy7 Biolegend Cat# 303433; RRID:AB_2734264; 1:100
Anti-human CD335 (NKp46) (clone 9 E2) PerCP Cy5.5 Biolegend Cat# 331920; RRID:AB_2561665; 1:50
Anti-human CD335 (NKp46) (clone 9 E2) PB Biolegend Cat# 331912; RRID: AB_2149280; 1:200
Anti-human CD40 (clone 5C3) BV421 Biolegend Cat# 334331; RRID:AB_AB_2564210; 1:50
Anti-human CD40 (clone 5C3) PE Biolegend Cat# 334308; RRID:AB_1186038; 1:50
Anti-human CD45 (clone HI30) PE Biolegend Cat# 304008; RRID:AB_2564156; 1:50
Anti-human CD45RA (clone HI100) BV 605 Biolegend Cat# 304134; RRID:AB_2563814; 1:400
Anti-human CD45RA (clone HI100) BV785 Biolegend Cat# 304139; RRID:AB_2563816; 1:100
Anti-human CD45RO (clone UCHL1) BV711 Biolegend Cat# 304235; RRID:AB_2562107; 1:25
Anti-human CD62L (clone DREG-56) APCCy7 biolegend Cat# 304813; RRID:AB_493583; 1:100
Anti-human CD66B (clone G10F5) PerCP Cy5.5 Biolegend Cat# 305108; RRID:AB_2077856; 1:50
Anti-human CD66B (clone G10F5) PB Biolegend Cat# 305112; RRID: AB_2563294; 1:200
Anti-human CD8 (clone RPA-T8) APCCy7 Biolegend Cat# 301016; RRID:AB_314133; 1:200
Anti-human CD80 (clone 2D10) BV421 Biolegend Cat# 305222; RRID:AB_2564407; 1:50
Anti-human LILRA4 (CD85g) (clone 17G10.2) Alexa647 biolegend Cat# 326410; RRID:AB_2265747; 1:25
Anti-human CD86 (clone 2331, FUN-1) BUV737 BD Biosciences Cat# 564428; RRID:AB_2738804; 1:50
Anti-human CLEC10A (clone H037G3) APC Biolegend Cat# 354705; RRID:AB_11218803; 1:50
Anti-human CELC10A (clone H037G3) PE Biolegend Cat# 354703; RRID:AB_11219202; 1:50
Anti-human DEC205 (clone 3G9) Alexa647 Obtained from Celldex Therapeutics, and labeled in-house; 1:2000

Anti-human HLA-DR (clone L243) APC Biolegend Cat# 307610; RRID:AB_314687; 1:100
Anti-human HLA-DR (clone L243) BV605 Biolegend Cat# 307640; RRID:AB_11219187; 1:100
Anti-human IFNAR1 (clone 85228) PE Thermo Fisher Cat# MA5-23630; RRID:AB_2609327; 1:50
Anti-human IFNAR2 (clone 122) FITC Thermo Fisher Cat# MA5-40953; RRID:AB_2898714; 1:50
Anti-human IFNAR2 (clone 122) APC Thermo Fisher Cat# MA5-40952; RRID:AB_2898713; 1:50
Anti-human IRF8 (clone V3GYWCH) PerCP-eFluor710 Thermo Fisher Cat#46-9852-82; RRID:AB_2573904; 1:400
Anti-human Ki67 (clone Ki-67) PerCP Cy5.5 Biolegend Cat# 350520; RRID:AB_2562295; 1:50
Anti-human SIRPa (CD172a) (clone SE5A5) PerCP Cy5.5 Biolegend Cat# 323811; RRID:AB_11219000; 1:100
Anti-human/mouse TCF4 (clone NCI-R159-6) Alexa647; Abcam Cat# ab246763; RRID:AB_2714172; 1:2000
Anti-human TNFR2 (clone 3G7A02) PE-Cy7 Biolegend Cat # 358411; RRID: AB_2564396; 1:50
Anti-human TNFR1 (clone W15099A) APC Biolegend Cat # 369905; RRID: AB_2650764 ; 1:50
Anti-mouse CD45 (clone 30-F11) BV785 Biolegend Cat# 103149; RRID:AB_2564590; 1:400
Anti-mouse CD45 (clone 30-F11) FITC Biolegend Cat# 103108; RRID:AB_312972; 1:200
Anti-mouse CD45 (clone 30-F11) PE Biolegend Cat# 103106; RRID:AB_312971; 1:800

Validation

All antibodies used were commercially available, well-validated clones routinely quality-controlled by their respective manufacturers. Prior to experimental use, each antibody was titrated to determine the optimal staining concentration under our specific assay conditions, including the different flow cytometry platforms used. Validation in our hands included confirming expected positive and negative populations based on well-characterized expression patterns. When used for the first time, fluorescence-minus-one (FMO) and isotype controls were included to define gating boundaries. Technical specifications and validation details for each antibody clone are available on the vendors' websites by referencing the catalog numbers listed above.

Plants

Seed stocks

Report on the source of all seed stocks or other plant material used. If applicable, state the seed stock centre and catalogue number. If plant specimens were collected from the field, describe the collection location, date and sampling procedures.

Novel plant genotypes

Describe the methods by which all novel plant genotypes were produced. This includes those generated by transgenic approaches, gene editing, chemical/radiation-based mutagenesis and hybridization. For transgenic lines, describe the transformation method, the number of independent lines analyzed and the generation upon which experiments were performed. For gene-edited lines, describe the editor used, the endogenous sequence targeted for editing, the targeting guide RNA sequence (if applicable) and how the editor was applied.

Authentication

Describe any authentication procedures for each seed stock used or novel genotype generated. Describe any experiments used to assess the effect of a mutation and, where applicable, how potential secondary effects (e.g. second site T-DNA insertions, mosiacism, off-target gene editing) were examined.

Flow Cytometry

Plots

Confirm that:

☒ The axis labels state the marker and fluorochrome used (e.g. CD4-FITC).

☒ The axis scales are clearly visible. Include numbers along axes only for bottom left plot of group (a 'group' is an analysis of identical markers).

☒ All plots are contour plots with outliers or pseudocolor plots.

☒ A numerical value for number of cells or percentage (with statistics) is provided.

Methodology

Sample preparation

Peripheral blood mononuclear cells (PBMCs) were isolated by density gradient centrifugation from buffy coats or blood of healthy donors using Ficoll-Paque PLUS (GE Healthcare), following manufacture instructions. Dendritic cells were isolated from fresh PBMCs by negative magnetic-bead enrichment followed by FACS-sorting. To enrich all mononuclear phagocytes, PBMCs were treated with human gamma-globulin (Invitrogen) for 15 minutes on ice to block non-specific binding and incubated with antibodies against CD3, CD19, CD335 and CD66b, followed by anti-mouse magnetic Dynabeads (ThermoFisher). Alternatively, PanDCs and pDCs were enriched using the EasySep Human PanDC pre-enrichment kit or EasySep Human Plasmacytoid DC Isolation Kit (STEMCELL), following manufacture instructions. For FACS purification, enriched cells were stained with antibody cocktail for 30 minutes on ice and sorted using FACSAria II or FACSAria Fuision (BD Biosciences). For naïve T cell preparation, frozen PBMCs were thawed, washed twice with PBS, incubated with 1.7 nM CFSE (Sigma-Aldrich) or 2.5 uM CellTrace Violet (ThermoFisher) at 37C in a water bath for 10 min, following by washing with R10 complete media. CD3+CD45RA+CD45RO- naïve T cells were isolated to >98% using EasySep Human Naïve Pan T cell isolation Kit (STEMCELL) according to manufacturer's instructions.

For in vitro culture, 10,000 sorted pDCs were cultured in 96-well U-bottom plates in 200ul R10 complete media [RPMI (Corning) with 10% heat-inactivated FBS (GIBCO), 2mM L-glutamine, 100IU/mL Penicillin, 100ug/mL Streptomycin, 10mM HEPES, 1mM Sodium Pyruvate, 1X MEM Nonessential Amino Acids (all Corning), and 55uM 2-Mercaptoethanol (GIBCO)] at 37C. All cultures contained 10ng/mL recombinant human IL-3 (R&D Systems; carrier-free) for pDC survival. Activation stimuli included 100-200ng/mL CD40L (R&D Systems; carrier-free), 2-2000ng/mL TNF (Biolegend; carrier-free), 10-1000U/mL IFNa (PBL Assay Science), or 5ug/mL CpG-A (ODN 2216, Invivogen). For IFN-I blockade, 1000ng/mL B18R (R&D) was added. For TNF blockade, pDCs were pre-incubated for 1 h with 10ug/mL anti-TNFR1 (clone 16805, R&D), anti-TNFR2 (clone 22210, R&D), or isotype control before CD40L stimulation. Secreted TNF and IL-8 were measured in day-1, 2 or 4 supernatants by Cytometric

Bead Array (CBA) Human Enhanced Sensitivity kit (BD Biosciences). For IFN-I detection, sorted DCs were cultured with 150uL R10 + IL-3 + 5ug/mL CpG-A for 24 h. Supernatants were frozen at -80C and analyzed with VeriKine Human IFN Alpha Multi-Subtype ELISA Kit (PBL Assay Science).

For flow cytometry, cells were then stained for 20 minutes at 4C with a cocktail of Abs against surface markers diluted in FACS buffer (2mM EDTA, 2% Donor equine serum in PBS), except the chemokine receptors CCR2 and CCR7 that were stained at 37C for 45 minutes in PBS. For Ki67, TCF4 and IRF8 staining, cells were stained with LIVE/DEAD Fixable Blue (ThermoFisher) in PBS for detection of dead cells, stained for surface markers, then fixed using Foxp3 Transcription Factor Fix/Perm Buffer (ThermoFisher) for 1 hr and stained intracellularly for 45 minutes in 1X Permwash buffer (ThermoFisher).

For CyTOF, fresh PBMCs and day-6 cultured pDCs were pooled with mouse splenocytes ("cell bed"), stained with 0.25mM cisplatin (Fluidigim), surface-stained with heavy-metal-labeled antibodies, fixed with Foxp3 Fix/Perm Buffer (ThermoFisher), and stained intracellularly. Cells were incubated overnight with 2% paraformaldehyde (Electron) in PBS with 125nM Iridium intercalator (Fluidigm), washed, filtered, and acquired in a CyTOF2 (Fluidigm) at the Shared FACS Facility at Stanford University.

For multiome sequencing data, DCs were sorted as live cells before nuclei isolation. Nuclei were extracted following the demonstrated protocol CG000365 adapted for low cell input nuclei from 10X Genomics. In brief, 100,000 FACS-sorted cells were washed with 0.04% BSA and treated with chilled multiome lysis buffer (10 mM Tris-HCl pH 7.4 (TEKnova), 10mM NaCl (Fisher Scientific), 3 mM MgCl2 (Sigma Adrich), 0.1% Tween-20 (Roche), 0.1% IGEPAL CA-630 (Sigma Aldrich), 0.01% Digitonin (Promega), 1% BSA (Sigma Aldrich), 1 mM DTT (ThermoFisher), 1 U/ul RNase inhibitor (Sigma Aldrich) prepared in nuclease-free water (Cytiva)) on ice for 3 min to obtain nuclei. The isolated nuclei were washed twice with multiome wash buffer (10 mM Tris-HCl pH 7.4, 10mM NaCl, 3 mM MgCl2, 0.1% Tween-20, 1% BSA, 1 mM DTT, 1 U/ul RNase inhibitor prepared in nuclease-free water), and then resuspended with chilled multiome nuclei buffer (1X Nuclei Buffer (20X) (10X Genomics), 1 mM DTT, 1 U/ul RNase inhibitor prepared in nuclease free water). Immediately after nuclei preparation, single-cell libraries were prepared at the Genomics core facility of Stanford University using Chromium Next GEM Single-Cell Multiome ATAC + Gene Expression kit (10X Genomics), and following the manufacturer's protocol. Libraries were sequenced on an Illumina NovaSeq instrument.

For SMARTseq2 data, cells were single cell FACS-sorted into lysis buffer. The Takara Smart-Seq Single Cell kit was used for reverse transcription, cDNA synthesis and amplification (Takara). For bulk sequencing, cells were sorted into lysis buffer for RNAseq or into media for ATACseq. RNA/DNA extraction and library preparation was performed at Stanford Functional Genomic Core.

For bulk RNA-seq, 10,000 pDCs from six adult (24-30 years) and six elderly (73-89 years) donors were sorted directly into Qiazol lysis buffer (Qiagen) and frozen at -80C for RNA sequencing. RNA extraction and library preparation were performed by the Stanford Functional Genomics Core. For ATAC-seq, 10,000 pDCs were sorted into R10, and DNA was extracted and libraries prepared using the Omni-ATAC protocol at the Stanford Functional Genomic Core. DNA was stored at -20C after transposition until all samples were collected. Amplification and qPCR were performed simultaneously across samples. Library quality was assessed by Bioanalyzer. The 24 samples were barcoded, pooled and sequenced on a NovaSeq 6000 at the Stanford Functional Genomics Core.

Instrument

Samples were analyzed on an LSR Fortessa X20 (BD Biosciences) or CYTEK Aurora (CYTEK). Sorting was performed on FACSAria II or Fusion (BD Biosciences).

Software

Data collection was performed with BD FACSDiva software (v8.01) or SpectroFlo software (v3.3.0). Data analysis was performed with FlowJo software v10.10.0 (Tree Star, Inc).

Cell population abundance

From each donor, approximately 50,000-100,000 cells were sorted from 50 mL of blood. Cell sorting gating strategies are shown in Extended Data Fig.1b, Extended Data Fig.2b, Extended Data Fig.5g, Extended Data Fig.6c, Extended Data Fig.10a., and Fig.2l Representative purification data is shown in Extended Data Fig.1b, Extended Data Fig.2b, Extended Data Fig.4b, Extended Data Fig.4e, Extended Data Fig.5h, Extended Data Fig.6c, Extended Data Fig.10a, and Fig.2l. Purity was >95% as determined by flow cytometry of postsort populations.

Gating strategy

Gating strategies for the analysis of DCs and other immune cells are shown in Extended Data Fig.1c, Extended Data Fig.4b, Extended Data Fig.4e, Extended Data Fig.4g, Extended Data Fig.5g-h, Extended Data Fig.6c, Extended Data Fig.8k, Extended Data Fig.10a, Extended Data Fig.10c, and Fig. 2c, Fig. 2d, Fig.2l, Fig.3i. Briefly, cells were gated on FSC/SSC, singlets, and live, prior to gate each population of interest. Positive gates were determined after titrating antibodies, using FMO or an isotype control antibody.

☒ Tick this box to confirm that a figure exemplifying the gating strategy is provided in the Supplementary Information.
